# Time-trial performance is not impaired in either competitive athletes or untrained individuals following a prolonged cognitive task

**DOI:** 10.1007/s00421-018-4009-6

**Published:** 2018-11-15

**Authors:** Ida E. Clark, Richie P. Goulding, Fred J. DiMenna, Stephen J. Bailey, Martin I. Jones, Jonathan Fulford, Sinead T. J. McDonagh, Andrew M. Jones, Anni Vanhatalo

**Affiliations:** 1 Sport and Health Sciences, College of Life and Environmental Sciences, St. Luke’s Campus, University of Exeter, Heavitree Road, Exeter, EX1 2LU UK; 2 School of Health Sciences, Liverpool Hope University, Hope Park Campus, Liverpool, Merseyside L16 9JD UK; 3 Division of Endocrinology, Diabetes and Bone Disease, Department of Medicine, Mt. Sinai St. Luke’s Hospital, New York, USA; 4 Department of Biobehavioral Sciences, Teachers College, Columbia University, New York, USA; 5 School of Sport, Exercise and Health Sciences, Loughborough University, Loughborough, UK

**Keywords:** Mental fatigue, Exercise performance, Anterior cingulate cortex, Cerebral oxygenation, Competitive athletes

## Abstract

It has been reported that mental fatigue decreases exercise performance during high-intensity constant-work-rate exercise (CWR) and self-paced time trials (TT) in recreationally-trained individuals. The purpose of this study was to determine whether performance is impaired following a prolonged cognitive task in individuals trained for competitive sport. Ten trained competitive athletes (ATH) and ten untrained healthy men (UNT) completed a 6-min severe-intensity CWR followed by a 6-min cycling TT immediately following cognitive tasks designed to either perturb (Stroop colour-word task and N-back task; PCT) or maintain a neutral (documentary watching; CON) mental state. UNT had a higher heart rate (75 ± 9 v. 69 ± 7 bpm; *P* = 0.002) and a lower positive affect PANAS score (19.9 ± 7.5 v. 24.3 ± 4.6; *P* = 0.036) for PCT compared to CON. ATH showed no difference in heart rate, but had a higher negative affect score for PCT compared to CON (15.1 ± 3.7 v. 12.2 ± 2.7; *P* = 0.029). Pulmonary O_2_ uptake during CWR was not different between PCT and CON for ATH or UNT. Work completed during TT was not different between PCT and CON for ATH (PCT 103 ± 12 kJ; CON 102 ± 12 kJ; *P* > 0.05) or UNT (PCT 75 ± 11 kJ; CON 74 ± 12 kJ; *P* > 0.05). Compared to CON, during PCT, UNT showed unchanged psychological stress responses, whereas ATH demonstrated increased psychological stress responses. However, regardless of this distinction, exercise performance was not affected by PCT in either competitive athletes or untrained individuals.

## Introduction

The factors responsible for the inability to sustain high-intensity exercise have long been debated (Contessa et al. 2016). Limiting factors might include a reduced capacity of the central nervous system (CNS) to activate required motor units and/or a decreased response of recruited muscle fibres to a given level of CNS activation (i.e. central or peripheral fatigue, respectively). However, a unifying concept is that a central or peripheral limitation of physiological origin creates the inability to maintain force/power output despite maximal voluntary effort. Challenges to this traditional model of exercise tolerance (e.g. Noakes et al. 2005) have been difficult to confirm empirically (Inzlicht and Marcora 2016).

Marcora et al. (2009) reported that time to exhaustion during high-intensity constant-work-rate (CWR) cycling was reduced following completion of a prolonged cognitive task (PCT) that required participants to engage in sustained attention, working memory, response inhibition and error monitoring for 90 min. Interestingly, this reduced ability to perform exercise was not accompanied by changes in mood, motivation or autonomic activation; however, subjective rating of perceived exertion (RPE) was increased throughout the exercise bout that followed the PCT. The authors concluded that ‘mental fatigue’ (MF) induced by the PCT heightened perception of physical effort independent of changes in cardiorespiratory, metabolic and/or neuromuscular responses (Marcora et al. 2009). This resulted in earlier disengagement from the exhaustive task and a corresponding inability to achieve the heart rate (HR) and blood lactate responses that were present at task failure when a neutral mental state was present (Marcora et al. 2009; Martin et al. 2018; Pageaux and Lepers 2016).

Perception of effort as a limiting factor in highly motivated participants challenges traditional notions regarding physiologically-based central and peripheral fatigue (Marcora and Staiano 2010). An ergolytic effect of MF on endurance performance may have implications for athletes both in competition and training. However, most research investigating the influence of MF on exercise performance has been conducted on individuals who performed regular physical training, but were not competitive athletes (Marcora et al. 2009; Brownsberger et al. 2013; Pageaux et al. 2013, 2014; MacMahon et al. 2014). One exception was a study involving professional road cyclists who did not experience the ergolytic effect of MF that was evident for recreational cyclists (Martin et al. 2016). Studies on non-professional competitive athletes have produced mixed results. For example, Van Cutsem et al. (2017a) found that male cyclists/triathletes did not experience impaired TT performance following MF; however, in that study, both MF and control conditions were performed in the heat (30 °C with 30% relative humidity). Consequently, the lack of effect of MF on TT performance could not be definitively ascribed to the athletic status of participants (Van Cutsem et al. 2017a).

The neurobiological link between prolonged performance of a cognitive task and impairment of exercise performance might reside in the anterior cingulate cortex (ACC) (Marcora et al. 2009), an area of the brain’s prefrontal cortex that is affected by MF (Lorist et al. 2005). Marcora et al. speculate that PCT affects the ACC’s effort-based decision making by increasing perception of effort during the exercise bout (Marcora et al. 2009; Pageaux et al. 2014, 2015; Smith et al. 2015, 2016). Interestingly, when subject perception of effort during exercise is decreased hypnotically with work rate held constant, the reduction in RPE is associated with decreased cerebral blood flow in this area of the brain (Williamson et al. 2001). While it is important to note that there is no evidence to suggest a causal relationship between RPE and ACC activity, it is possible that an alteration in ACC activity could be an objectively measurable link between PCT and decreased exercise performance (Marcora et al. 2009).

The purpose of this study was to determine the influence of PCT on exercise performance in competitive athletes. We asked athletes involved in a variety of sports (ATH) and untrained participants (UNT) to perform 6 min of CWR exercise followed by a 6-min TT both with (PCT) and without (CON) completion of a 30-min PCT protocol that required response inhibition. We hypothesised that PCT would decrease the amount of work completed during TT in UNT, but not ATH. During both the PCT and the exercise bout comprising CWR and TT, we used near-infrared spectroscopy (NIRS) to assess cerebral oxygenation as a way to infer frontal-lobe brain activation (Ehlis et al. 2005; Sakatani et al. 1999; Schroeter et al. 2002). We hypothesised that frontal-lobe activation would be greater in the PCT compared to control condition for UNT, but not ATH.

## Methods

### Participants

Ten competitive male athletes (mean ± SD: age 27.4 ± 6.3 year; stature 1.79 ± 0.06 m; body mass 75.6 ± 9.7 kg) and ten healthy males who did not participate in structured physical activity (age 25.8 ± 4.6 year; stature 1.83 ± 0.06 m; body mass 93.3 ± 17.0 kg) were recruited to participate in this study, which was approved by the University of Exeter Research Ethics Committee. The ATH group comprised three cyclists, two triathletes, two footballers, a distance runner, a crossfit athlete and a boxer. All participants were required to provide written informed consent, complete a medical-health questionnaire and provide an exercise log to assess training status prior to participating in the study. The total time spent exercising per week at low, moderate, high and very-high intensities was calculated for each participant and training status was quantified using a physical-activity rating tool (PA-R). The ATH group trained > 9.5 h per week and had a PA-R score of 9.7 (range 8–10), whereas the UNT group did < 3 h of physical activity per week and had a PA-R score of 3.8 (range 1–6). Participants were instructed to refrain from alcohol ingestion or strenuous exercise participation for 24 h prior to each laboratory visit and to arrive at the laboratory in a rested and fully hydrated state.

### Experimental overview

**Fig. 1:**
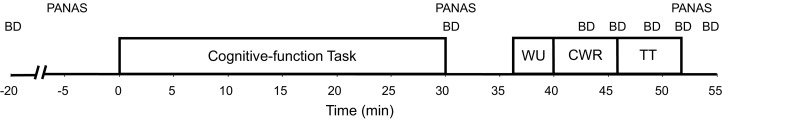
Schematic illustration of the experimental protocol during laboratory visits three and four. A 15-min exercise bout comprising a 3-min warm-up (WU), 6-min constant-work-rate bout at 70%∆ (CWR) and 6-min time trial (TT) was initiated 7 min following completion of a cognitive task that either perturbed (experimental condition) or maintained (control condition) a neutral mental state. The times at which blood was drawn for sampling (BD) and positive and negative affect were assessed (PANAS) are indicated

The participants reported to the laboratory on four occasions at the same time of day (± 2 h) with visits separated by ≥ 24 h. All participants completed all testing sessions in 2–4 weeks. During the first visit, participants completed a ramp incremental cycling test to the limit of tolerance to assess cardiorespiratory fitness. The second visit was used to familiarise participants with the procedures required for the two exercise trial sessions, which comprised visits three and four. At the beginning of these sessions, an intravenous cannula was inserted in the antecubital vein of the subject’s arm to permit blood sampling throughout the exercise bout. The order in which these exercise sessions were performed was randomised. A schematic representation of the timeline of events in sessions 3 and 4 is provided in Fig. 1.

### Cognitive tasks

Both exercise trial sessions began with participants performing cognitive tasks designed either to perturb (PCT) or maintain (CON) a neutral mental state. The PCT comprised 30 min of performance of a computer-based modified version of the Stroop colour-word task and N-back task (E-Prime 2.0; Psychology Software Tools, Inc. 2013). The Stroop colour-word and N-back tasks were alternated on a 3- and 2-min loop, respectively. Participants were seated comfortably at a desk in the laboratory beside the cycle ergometer. The computer monitor was positioned at eye level and a keyboard with four colour-coded keys (red, green, blue and yellow) was placed in front of the participant who was wearing both ear plugs and noise-cancelling headphones to eliminate auditory distraction.

The Stroop colour-word task involved the presentation of text with stimuli classified as: (1) ‘congruent’ where a word describing a colour (‘Red’, ‘Green’, ‘Blue’ or ‘Yellow’) was presented with the text written in the same colour as the word; (2) ‘incongruent’ where the same words were presented, but the colour of the text did not match the word (e.g. the word ‘Red’ was written with blue font); or (3) neutral where ‘XXX’ written in one of the font colours used for the congruent and incongruent conditions was presented. Each text item was presented for 1700 ms followed by a blank screen for 700 ms with the stimuli balanced such that an equal number of congruent, incongruent and neutral items were presented. Subjects were instructed to push the key which indicated the colour in which the word was presented. The N-back task involved the display of a series of single letters presented on the screen one at a time for 1000 ms followed by a blank screen for 750 ms. If the letter on display was the same as the letter presented two letters previously (2-back protocol), subjects were instructed to press the green key. If the letter was different, subjects were instructed to press the blue key. Labels indicating ‘yes’ or ‘no’ were placed above the green and blue key, respectively. A one-minute practice session comprising 30 s of performance of each cognitive task was allowed prior to beginning the trial to ensure understanding. Subjects were told that they were being tested on both response accuracy and reaction time, which were each recorded and averaged for 5-min bins for the entire PCT (Fig. 1).

The CON condition consisted of watching Episode 5 of ‘The Life of Birds’ documentary by Sir David Attenborough for 30 min. The purpose was to establish a ‘neutral mood’ for the subject (Wirth et al. 2011). Participants were seated in the same chair at the same desk as during the PCT. Participants also wore the same noise-cancelling headphones; however, in this case, the soundtrack of the documentary was provided for auditory engagement.

### Exercise tasks

#### Ramp incremental cycling test

On the first laboratory visit, participants completed a ramp incremental cycling test for determination of the gas exchange threshold metabolic rate (GET_met_) and peak rates of oxygen uptake and work ($\dot{V}{\text{O}}_{{2{\text{peak-INC}}}}$ and *W*_peak-INC,_ respectively). This test was performed on an electronically braked cycle ergometer (Lode, Excalibur, Groningen, The Netherlands) with seat height and handles adjusted for comfort. The settings were recorded so that the setup could be replicated in subsequent tests. Participants began with 3 min of baseline cycling at 20 W after which the work rate was increased in a continuous manner at 25 (UNT) or 35 (ATH) W per min (i.e. 1 W per 2.4 or 1.7 s, respectively) until the limit of tolerance was reached. Participants were instructed to cycle at 80 rpm throughout with the test terminated when the pedal rate fell by > 5 rpm for more than 5 s despite strong verbal encouragement. Breath-by-breath pulmonary gas-exchange data were collected continuously throughout the test and averaged into 10-s bins. The $\dot{V}{\text{O}}_{{2{\text{peak - INC}}}}$ was defined as the highest 30-s rolling-average value during the test. The GET_met_ was estimated using a cluster of measures including: (1) the first disproportionate increase in the rate of carbon dioxide output ($\dot{V}{\text{CO}}$) from visual inspection of individual plots of $\dot{V}{\text{CO}}_{2}$ versus $\dot{V}{\text{O}}_{2}$; (2) an increase in minute ventilation ($\dot{V}_{E}$) relative to $\dot{V}{\text{O}}_{2}$ with no increase in $\dot{V}_{E}$ relative to $\dot{V}{\text{CO}}_{2}$; and (3) an increase in end-tidal O_2_ tension with no fall in end-tidal CO_2_ tension. For each incremental test, GET_met_ was estimated independently by experienced examiners and a consensus estimate was established. To align GET_met_ with the work rate that precipitated it (*W*_GET_), account was made for the mean response time of the $\dot{V}{\text{O}}_{2}$ response, which was estimated as 40 s (Whipp et al. 1981) (i.e. ~17 and ~23 W of work for UNT and ATH, respectively). The CWR bouts completed in subsequent laboratory sessions were completed with the work rate set at 70%∆ (*W*_GET_ plus 70% of the difference between *W*_GET_ and *W*_peak-INC_).

#### Constant-work-rate cycling test and cycling time trial

On the third and fourth laboratory visits, participants performed a CWR/TT cycling bout that was initiated 7 min following completion of the cognitive task. The CWR portion of the bout began with 3 min of warm-up cycling at 20 W. Following this baseline period, work rate was increased in a step fashion to 70%∆ (see above) after which participants cycled for 6 min. Participants were instructed to maintain cadence at 80 rpm during this bout. Immediately following completion of 6 min of CWR cycling, the ergometer mode was changed from hyperbolic to linear so that the subject could perform a 6-min TT during which they were instructed to complete as much work as possible. To ensure consistency across subjects, resistance on the pedals during the TT was set specifically so that they would attain their power output at 70%Δ when pedalling at 80 rpm (linear factor = power/cadence^2^). To aid in TT performance, a clock was visible so that participants were aware of the time remaining. The same investigator was present at each testing session and similar verbal encouragement was provided every 30 s to ensure consistent motivation across experimental conditions. Power output was measured continuously throughout the TT and the total work completed (Work_TT_) was calculated in kilojoules by multiplying the mean power output in watts by 360 s. Bin-averaged 30-s power outputs were also calculated for the 6-min TT.

### Physiological measures

#### Metabolic data

During all exercise tests, pulmonary gas exchange and ventilation were measured breath by breath using an online gas analyzer (Jaeger Oxycon Pro, Hoechberg, Germany). Participants wore a nose clip and breathed through a low-dead-space, low-resistance mouthpiece and an impeller turbine assembly (Jaeger Triple V). A capillary line was connected to the mouthpiece and inspired and expired gas volume and gas concentration signals were sampled continuously at 100 Hz using paramagnetic (O_2_) and infrared (CO_2_) analyzers (Jaeger Oxycon Pro, Hoechberg, Germany). The gas analyzers were calibrated before each test with gases of known concentration and the turbine volume transducer was calibrated using a 3-L syringe (Hans Rudolph, Kansas City, MO). The volume and concentration signals were time aligned by accounting for the delay in capillary gas transit and analyser rise time relative to the volume signal.

Total O_2_ consumed was determined for the CWR bout by integrating the area under the $\dot{V}{\text{O}}_{2}$/time curve for 0–120 s, 0–180 s and the entire 360 s. Total O_2_ consumed during the entire TT was determined in a similar manner. The peak $\dot{V}{\text{O}}_{2}$ during CWR ($\dot{V}{\text{O}}_{2peak-CWR}$) and TT ($\dot{V}{\text{O}}_{2peak-TT}$) were defined as the highest 10-s mean $\dot{V}{\text{O}}_{2}$ values from 0 to 360 s and 360 to 720 s of the CWR and TT bout.

#### Heart rate

Subjects’ HR was measured continuously during all exercise tests using short-range radiotelemetry (Polar S610; Polar Electro Oy, Kempele, Finland) with a sampling frequency of 5 s. The mean HR was calculated for the final 60 s of baseline cycling and for 0–120 s, 120–240 s and 240–360 s of CWR. The mean HR during TT was determined in a similar manner and the average HR during each 30-min cognitive task was also calculated.

#### Cerebral oxygenation

The oxygenation of the frontal lobe was monitored using NIRS (NIRO 300; Hamamatsu Photonics KK, Hiugashi-ku, Japan). The NIRS probe consisted of a rubber holder containing a detector and an emitter separated by 4 cm. Double-sided tape was attached to the probe, which was adhered 3 cm above the left eyebrow (i.e. between Fp1 and F3 of the prefrontal cortex; PFC) according to the modified International EEG 10–20 system (Perrey 2008). The NIRS data were collected at a sampling frequency of 2 Hz and the Beer–Lambert law was used to calculate changes in the concentration of tissue oxyhemoglobin (∆[HbO_2_]) and deoxyhemoglobin (∆[HHb]) using optical densities and a differential path length factor of 5.93 (Duncan et al. 1995). The ∆[HbO_2_] and ∆[HHb] were summed to provide an estimate of total blood volume (∆[Hb_tot_]) and tissue oxygenation index (TOI) was also calculated. The NIRS measurement began with 60 s of resting data collection prior to the cognitive task and subsequent changes were calculated relative to this baseline. The TOI was expressed as a percentage. The ∆[HbO_2_], ∆[HHb], ∆[Hb_tot_] and TOI data collected during the cognitive tasks and CWR/TT bouts were averaged into 300- and 120-s bins, respectively, and overall exercise values were also determined by averaging the data for the entire 360 s of both CWR and TT.

#### Blood sampling

Venous blood samples were collected into lithium-heparin (LH) and ethylenediaminetetraacetic-acid (EDTA) vacutainers (Becton–Dickinson, New Jersey, USA) on seven occasions throughout laboratory sessions three and four (Fig. 1). Immediately after sampling, 200 µL of blood was extracted from the LH vacutainer into 200 µL of Triton-X-100 solution (Triton X-100, Amresco, Salon, OH) and plasma lactate and glucose concentrations were measured ([lactate] and [glucose], respectively) (YSI 1500; Yellow Springs Instrument, Yellow Springs, OH). The remaining blood was centrifuged at 4000 rpm for 8 min at 4 °C after which plasma was extracted and analyzed for potassium and sodium concentrations ([K^+^] and [Na^+^], respectively) (9180 Electrolyte Analyzer, F. Hoffmann-La Roche, Basel, Switzerland). The plasma obtained from the EDTA vacutainers was frozen at − 80 °C and, upon completion of the study, defrosted at room temperature and analysed for cortisol concentration ([cortisol]) using an ELISA kit (Abnova, Taiwan).

#### Psychological measures

Subject affect was assessed using the positive and negative affect scale (PANAS; Watson et al. 1988). This was administered before and immediately following the cognitive tasks and 1 min after completion of TT (Fig. 1). Participants were instructed to answer the questions as they were feeling ‘right now’. The questionnaire consisted of 20 words that describe positive (*n* = 10; e.g. ‘excited’, etc.) and negative (*n* = 10; e.g. ‘irritable’, etc.) feelings presented in random order. Participants were instructed to record a number between one and five (1 = very slight or not at all; 2 = a little; 3 = moderately; 4 = quite a bit; 5 = extremely) next to each word. The negative and positive scores were then summed separately. Total scores for positive and negative affect each ranged from 10 to 50.

### Statistical analysis

Statistical analysis was conducted using SPSS version 22.0 (SPSS Armonk, NY) and all data are reported as mean ± SD. Across-group comparisons for $\dot{V}{\text{O}}_{{2{\text{peak--INC}}}}$, *W*_peak-INC,_*W*_GET_, GET_met_, power output at 70%∆, PA-R and total exercise time per week in the various intensity zones were made using paired-sample *t* tests. A 2 × 2 (condition × group) repeated-measures ANOVA (RMANOVA) was used to compare the mean HR response during the cognitive tasks and *W*_TT_ while a 2 × 6 (group × time) RMANOVA was used to compare PCT response accuracy and reaction time. A 2 × 2 × 3 (condition × group × time) RMANOVA was employed to compare PANAS positive- and negative-affect measurements and the ∆[HbO_2_], [∆HHb], [∆Hb_tot_] and TOI overall exercise responses while a 2 × 2 × 6 RMANOVA was used to compare the 300- and 120-s mean responses for ∆[HbO_2_], [∆HHb], [∆Hb_tot_] and TOI during the cognitive tasks and CWR/TT, respectively. Total O_2_ consumed during each 120 s of CWR/TT was also analysed using a 2 × 2 × 6 RMANOVA. Plasma [cortisol] measurements for pre cognitive task, post cognitive task, end-TT and 3-min-post end-TT were compared using a 2 × 2 × 4 RMANOVA and RMANOVA was also used to analyse plasma [lactate], [glucose], [K^+^] and [Na^+^] measurements at the same time points in addition to mid-CWR, end-CWR and mid-TT (2 × 2 × 7). In addition, a 2 × 2 × 7 RMANOVA was used to compare HR and *V̇*O_2_ measurements for the time bins averaged for baseline and throughout the exercise. Finally, 30-s mean power outputs during TT were analysed using a 2 × 2 × 12 RMANOVA. In all cases, when the sphericity assumption was violated, the Greenhouse–Geisser correction was employed and post-hoc tests were performed using pairwise comparison with Bonferroni correction. Statistical significance was accepted when *P* < 0.05.

## Results

### Participant characteristics

Between-group comparisons for $\dot{V}{\text{O}}_{{2{\text{peak--INC}}}}$, *W*_peak-INC,_*W*_GET_, PA-R and total exercise time per week are provided in Table 1. The $\dot{V}{\text{O}}_{{2{\text{peak--INC}}}}$, *W*_peak-INC_, *W*_GET_ and PA-R were all significantly greater for ATH compared to UNT (*P* < 0.01). Furthermore, compared to UNT, ATH performed a significantly greater amount of exercise per week at high and very-high intensities. The power output at 70%∆ that was maintained during CWR was greater for ATH compared to UNT (325 ± 34 v. 224 ± 24 W; *P* < 0.001). Due to technical difficulties, data collected during the PCT for one member of the ATH group were excluded from these results.

**Table 1:** Fitness characteristics for the athletes and untrained subjects who were assessed in this study

	ATH	UNT
$\dot{V}{\text{O}}_{{2{\text{peak--INC}}}}$ (mL kg^−1^ min^−1^)	58.3 ± 4.1	39.0 ± 7.3*
*W*_peak-INC_ (W)	401 ± 36	280 ± 27*
*W*_GET_ (W)	149 ± 32	91 ± 25*
PA-R	9.7 ± 0.7	3.8 ± 2.2*
Total exercise time (h week^−1^)	9.5 ± 2.5	3.2 ± 1.9*
Low-intensity exercise time (h week^−1^)	0.5 ± 0.8	1.2 ± 2.2
Moderate-intensity exercise time (h week^−1^)	2.3 ± 3.1	1.2 ± 1.8
High-intensity exercise time (h week^−1^)	3.8 ± 1.4	0.8 ± 0.9*
Very-high-intensity exercise time (h week^−1^)	2.9 ± 1.7	0.0 ± 0.0*

Values are presented as mean ± SD

$\dot{V}{\text{O}}_{2peak-INC}$ peak rate of oxygen uptake during incremental test, *W*_*peak-INC*_ peak rate of work during incremental test, *W*_*GET*_ estimated rate of work at gas exchange threshold, *PA-R* physical-activity rating

*Significantly different from ATH (*P* < 0.01)

### Psychological responses during cognitive tasks

**Fig. 2:**
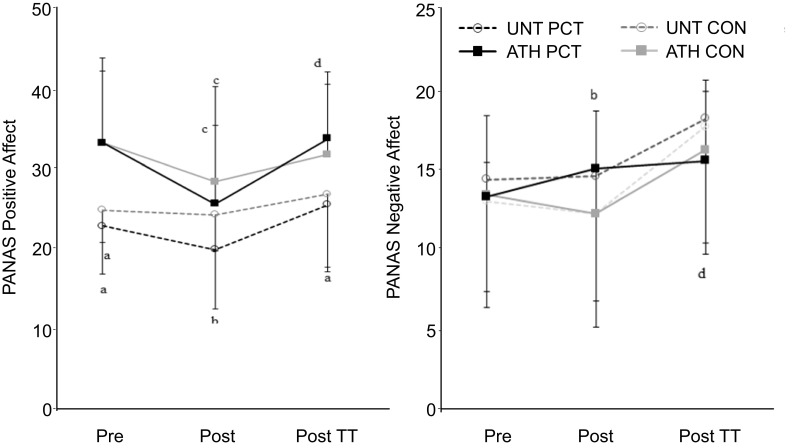
Positive (left panel) and negative (right panel) affect ratings for competitive athletes (ATH) and untrained participants (UNT) pre and post task that either perturbed (PCT) or maintained (CON) a neutral mental state. Post time trial (TT) values are also provided for comparison. Values are mean ± SD. **a** Significant difference compared to ATH group (*P* < 0.05); **b** significant difference compared to CON condition (*P* < 0.05); **c** significant difference compared to baseline value (*P* < 0.05); **d** significant difference compared to post-cognitive-task value (*P* < 0.05)

The results of the PANAS assessment before and after the cognitive tasks are depicted in Fig. 2. For positive affect, UNT had a significantly lower value following PCT compared to CON (19.9 ± 7.5 v. 24.3 ± 4.6; *P* = 0.036) while for negative affect, ATH had a significantly higher value following PCT compared to CON (15.1 ± 3.7 v. 12.2 ± 2.7; *P* = 0.029).

### Physiological responses during cognitive tasks

Compared to ATH, UNT had a higher mean HR during both cognitive tasks (PCT, 75 ± 9 v. 59 ± 6 b min^−1^; CON, 69 ± 7 v. 58 ± 6 b min^−1^; *P* < 0.005 in both cases). A between-condition difference was also present for UNT who demonstrated a higher HR during PCT compared to CON (*P* = 0.002). Conversely, there was no difference in mean HR between PCT and CON for ATH. There were no between-group or between-condition differences in plasma [lactate], [glucose], [K^+^], [Na^+^] or [cortisol] before or after the cognitive tasks. There were also no changes in any of the blood variables during the cognitive tasks except for [K^+^], which increased slightly for UNT during PCT (post 4.4 ± 0.5 mM; pre 3.9 ± 0.30 mM; *P* = 0.002).

### Cerebral-oxygenation responses during cognitive tasks

There were no between-group differences for cerebral ∆[HbO_2_], ∆[HHb] or ∆[Hb_tot_] at any time point during either cognitive task; however, UNT had a higher TOI at all time points during both PCT and CON. Furthermore, for UNT, TOI was greater than baseline for all time points beyond 10 min during both cognitive tasks, whereas for ATH, TOI did not change. There were no between-condition differences for any of the cerebral-oxygenation variables for either group at any time point during the cognitive tasks.

### Performance during prolonged cognitive task

Response accuracy and reaction time did not change significantly over time during the PCT for either group. Furthermore, there were no between-group differences for response accuracy at any time point during the 30-min PCT task; however, UNT had a significantly shorter response time compared to ATH at all time points other than the final 5 min.

### Psychological responses during exercise

The results of the PANAS assessment following the TTs are shown in Fig. 2. Compared to post-cognitive task, post-exercise positive affect was significantly increased for ATH (*P* = 0.033) whereas post-exercise negative affect was significantly increased for UNT (*P* = 0.012). No between-condition differences were present for post-exercise positive or negative affect for either group.

### Physiological responses during exercise

Group-by-condition comparisons for $\dot{V}{\text{O}}_{2}$ variables and total O_2_ consumed during the CWR/TT bout under PCT and CON conditions are shown in Table 2. There were no significant differences between PCT and CON for any metabolic variables for ATH, whereas for UNT, $\dot{V}{\text{O}}_{{2{\text{peak--TT}}}}$ was lower in PCT compared to CON (*P* = 0.04). There were no between-condition differences for $\dot{V}{\text{O}}_{2}$ or HR during any of the time bins averaged during CWR or TT.

**Table 2:** Metabolic data for athletes (ATH) and untrained subjects (UNT) during exercise performed after cognitive tasks that perturbed (PCT) or maintained (CON) a neutral mental state

	ATH		UNT	
	PCT	CON	PCT	CON
$\dot{V}{\text{O}}_{\text{2base}}$ (L min^−1^)	1.13 ± 0.11	1.07 ± 0.23	1.14 ± 0.10	1.17 ± 0.13
$\dot{V}{\text{O}}_{{2{\text{peak--CWR}}}}$(L min^−1^)	4.48 ± 0.45	4.46 ± 0.41	3.35 ± 0.33*	3.44 ± 0.35*
$\dot{V}{\text{O}}_{{2{\text{peak--TT}}}}$(L min^−1^)	4.50 ± 0.46	4.49 ± 0.50	3.55 ± 0.29*	3.64 ± 0.32*^†^
Total O_2_ consumed 0–120 s (L)	4.9 ± 0.4	4.9 ± 0.4	3.4 ± 0.5*	3.4 ± 0.6*
Total O_2_ consumed 0–180 s (L)	8.8 ± 0.7	8. 8 ± 0.7	6.1 ± 0.9*	6.3 ± 0.9*
Total O_2_ consumed 0–360 s (L)	21.6 ± 1.6	21.5 ± 1.7	15.6 ± 2.1*	15.9 ± 2.1*
Total O_2_ consumed TT (L)	25.2 ± 2.2	25.1 ± 2.0	19.2 ± 2.4*	19.6 ± 2.5*

Values are presented as mean ± SD

$\dot{V}{\text{O}}_{2base}$ rate of oxygen uptake during baseline cycling, $\dot{V}{\text{O}}_{2peak-CWR}$ peak rate of oxygen uptake during the constant-work-rate bout, $\dot{V}{\text{O}}_{2peak-TT}$ peak rate of oxygen uptake during the time trial

*Significantly different from ATH within condition (*P* < 0.05)

^†^Significantly different from PCT within group (*P* < 0.05)

Group-by-condition comparisons for plasma [lactate], [glucose] and [cortisol] at baseline, end-TT and 3-min-post end-TT are provided in Table 3. Plasma [lactate] increased from baseline in a similar manner during exercise for both groups during both conditions, whereas there was no change in plasma [cortisol] from baseline during exercise for either group during either condition. However, 3 min following the exercise bout, plasma [cortisol] was significantly greater compared to immediately post-exercise for UNT regardless of the cognitive task, whereas for ATH, this was only the case for CON. There were no other temporal changes or between-condition differences for any of the blood-sampling variables during or post-exercise except for [K^+^] and [Na^+^], which each increased from baseline in a similar manner during exercise for both groups during both conditions.

**Table 3:** Blood-sampling data for athletes (ATH) and untrained subjects (UNT) before and following exercise performed after cognitive tasks that perturbed (PCT) or maintained (CON) a neutral mental state

	ATH		UNT	
	PCT	CON	PCT	CON
Baseline [lactate] (mM)	0.8 ± 0.2	1.0 ± 0.3	1.2 ± 0.5	1.0 ± 0.5
End-exercise [lactate] (mM)	10.7 ± 2.1	10.2 ± 1.7	8.8 ± 1.9	8.6 ± 1.7
Three-min post [lactate] (mM)	10.7 ± 2.0	8.9 ± 2.8†	9.6 ± 1.8	9.7 ± 2.3
Baseline [glucose] (mM)	4.2 ± 0.5	4.8 ± 1.3	4.6 ± 1.2	4.5 ± 0.8
End-exercise [glucose] (mM)	4.5 ± 0.7	4.6 ± 0.7	4.1 ± 1.0	3.8 ± 0.9
Three-min post [glucose] (mM)	5.7 ± 1.0	5.0 ± 1.5	5.2 ± 1.8	4.6 ± 1.0
Baseline [cortisol] (µg dL^−1^)	11.5 ± 6.2	10.4 ± 5.4	12.5 ± 4.3	12.4 ± 2.8
End-cognitive-task [cortisol] (µg dL^−1^)	10.3 ± 3.4	8.8 ± 2.8	10.0 ± 2.1	9.9 ± 2.3
End-exercise [cortisol] (µg dL^−1^)	10.0 ± 3.5	8.6 ± 2.5	10.4 ± 2.7	10.5 ± 2.5
Three-min post [cortisol] (µg dL^−1^)	10.6 ± 3.2	10.0 ± 2.4	12.1 ± 2.6	12.8 ± 2.7*

Values are presented as mean ± SD

*Significantly different from ATH within condition (*P* < 0.05)

^†^Significantly different from PCT within group (*P* < 0.05)

### Cerebral-oxygenation responses during exercise

**Fig. 3:**
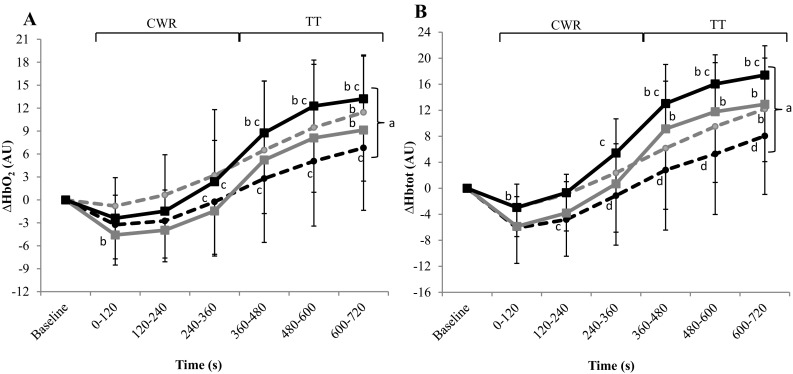
Cerebral ∆HbO_2_ (left panel) and ∆HbO_tot_ (right panel) for competitive athletes (squares) and untrained subjects (circles) prior to and during a 12-min bout of constant-work-rate (CWR) and time-trial (TT) cycling following cognitive-function tasks that either perturbed (black) or maintained (gray) a neutral mental state. **a** Significant main effect of time (*P* < 0.001). **b** Significantly different from baseline value (*P* < 0.05). **c** Significantly different from CON (*P* < 0.05). **d** Significantly different from ATH (*P* < 0.05)

The mean ± SD values for cerebral-oxygenation responses during CWT/TT are depicted for each group in Fig. 3. For ATH, there were increases in the ∆[HbO_2_], ∆[HHb] and ∆[Hb_tot_] responses from CWR to TT in both PCT and CON (*P* < 0.001). Increases in ∆[HbO_2_] and ∆[Hb_tot_] were also present in the transition from CWR to TT for UNT in PCT and CON (*P* < 0.001), but in UNT the ∆[HHb] decreased significantly during CWR and increased during the TT in the PCT condition (*P* < 0.05). There was a between-group difference for TOI with UNT having a lower overall exercise response compared to ATH (63 ± 9 v. 71 ± 8%, *P* < 0.05). Moreover, the overall ∆[HbO_2_] response during exercise was greater during PCT compared to CON for ATH but greater during CON compared to PCT for UNT (*P* = 0.025 and 0.028, respectively). Specifically, ATH had a greater ∆[HbO_2_] for the PCT condition during both CWR and TT, whereas UNT had a reduced response with PCT during TT. ATH also had a greater ∆[Hb_tot_] for PCT compared to CON during both CWR and TT whereas there was no between-condition difference for UNT during either form of exercise.

### Performance during the TT

**Fig. 4:**
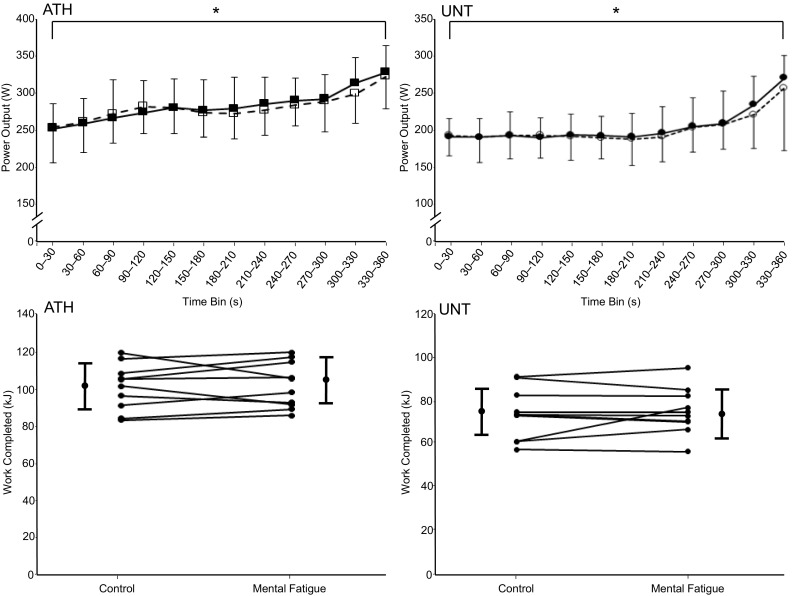
Thirty-second average power outputs for competitive athletes (left top panel) and untrained participants (right top panel) throughout the 6-min time trial that followed a cognitive-function task that either perturbed (closed squares/circles) or maintained (open squares/circles) a neutral mental state. Values are mean ± SD. *Main effect of time (*P* < 0.05). Notice how the self-regulated pacing profile was not altered by PCT in either group. Furthermore, as illustrated by the individual-subject and group mean ± SD data depicted in the lower panels, the work completed during the time trial for the competitive athletes (left; *n* = 10) and untrained participants (right; *n* = 10) was also unaffected by PCT

The 30-s mean power outputs throughout TT are depicted in Fig. 4 (top panels). Power output increased significantly during TT for ATH and UNT; however, there were no between-condition differences in power output at any time point during TT in either group. There were also no between-condition differences for work completed during TT for either group (Fig. 4, bottom panels).

## Discussion

The main original finding of this investigation was that highly-trained competitive athletes did not experience a decline in self-paced TT performance when exercise was performed shortly after a prolonged challenging cognitive task. This confirms the initial part of our first hypothesis and is consistent with the notion that trained competitive athletes possess characteristics that are resilient to the potential/putative adverse effect of PCT on exercise performance. However, contrary to the second part of our first hypothesis, the exercise performance of UNT was not adversely affected by PCT. Furthermore, with respect to cortical activation, NIRS measurements suggested a greater frontal-lobe activity for ATH during exercise after PCT, which is in contrast to our second hypothesis. Collectively, these findings indicate complexity in the possible ergolytic effects of PCT during self-regulated exercise.

In the present study, we chose to assess the influence of PCT on exercise performance in two distinctly different groups of individuals based on their experience with competitive sport and exercise training. Our objective was to determine if athletic status influences the degree to which PCT may impair exercise performance. Most previous investigations have involved assessment of a single group of participants with similar fitness characteristics (for overview, see Table 5 by Van Cutsem et al. 2017b; cf. Martin et al. 2016). Indeed, most of the research that has identified an ergolytic effect of PCT has involved individuals who performed regular physical training, but not competitive sport (Marcora et al. 2009; Brownsberger et al. 2013; Pageaux et al. 2013, 2014; MacMahon et al. 2014). In investigations that have assessed the influence of PCT in competitive athletes, results have been mixed (Duncan et al. 2015Martin et al. 2015; Martin et al. 2016; Smith et al. 2016; Van Cutsem et al. 2017a). In the only study on professional endurance athletes, Martin et al. (2016) reported that PCT did not alter perception of effort or performance of road cyclists during a 20-min TT. However, an ergolytic effect of PCT was observed for recreational cyclists in that study (Martin et al. 2016) with other studies confirming such an effect for soccer players (Smith et al. 2016) and team-sport athletes (Smith et al. 2015) during intermittent running. In other studies, PCT did not affect ‘anaerobic’ performance in team-sport players (Duncan et al. 2015) or mean power output during the final 30 s of all-out cycling for either team-sport players or triathletes (Martin et al. 2015). With respect to the latter finding, this ‘end-test power’ approximates the critical power (CP; Vanhatalo et al. 2007), a parameter that reflects the greatest metabolic rate that can be sustained without progressive increase in muscle metabolic perturbation (Poole et al. 2016). Moreover, work completed above end-test power during the all-out bout was also unaffected by PCT (Martin et al. 2015). As these two parameters collectively dictate time to exhaustion during high-intensity exercise (Poole et al. 2016), this implies that high-intensity endurance performance (e.g. CWR time to task failure) would not have been altered had it been assessed. Finally, PCT did not impair cycling TT performance for cyclists/triathletes in the heat (Van Cutsem et al. 2017a). While these disparate findings might reflect differences in the cognitive challenge used to perturb mental state and/or different characteristics of the criterion exercise challenge (e.g. CWR exercise or TT, continuous or intermittent, intensity and/or duration) our results appear to cohere with those of Martin et al. (2016) for professional athletes and suggest that competitive athletes do not experience an ergolytic effect of PCT during endurance-exercise performance.

One factor that should be considered when interpreting the present findings is that the 6-min cycling TT we chose for performance assessment was different from those that have been used in the past for PCT research. For example, MacMahon et al. (2014) had participants perform a 3-km running TT that required ~ 12 min to complete, Pageaux et al. (2014) had participants perform a 5-km running TT, and Martin et al. (2016) had participants run for a constant duration of 20 min. In addition to employing a different exercise modality, we opted for a shorter TT to improve reliability (Currell and Jeukendrup 2008); however, the duration we chose should still be long enough to result in a high proportional contribution of oxidative contribution to total energy turnover. This is important because PCT appears to exert no effect on ‘anaerobic’ performance (Duncan et al. 2015).

In the present study, we also studied untrained individuals to provide a contrast with respect to the athletic and training experience of the participant. We reasoned that UNT would provide the ideal control group against which to compare the influence of PCT in ATH because they would presumably be most susceptible to a potential adverse effect on TT performance. However, contrary to our hypothesis, UNT also showed no reduction in work performed or change in pacing profile when exercising following PCT. It is important to note that inclusion of UNT qualifies ours as the first study to investigate the effect of PCT on exercise performance in participants with an ‘average’ cardiorespiratory fitness level (e.g. $\dot{V}{\text{O}}_{2peak}$, ~ 40 mL kg^−1^ min^−1^) and limited training experience. This raises the intriguing possibility that the psychological stress associated with unfamiliar intense exercise might have served as a cognitive stimulus for UNT that was mechanistically similar to (and, therefore, with respect to ergolytic effect, not additive with) that which was elicited by PCT. In this regard, it is interesting to note that our two groups demonstrated markedly different patterns of two-dimensional affect response during the exercise challenge. Specifically, for ATH experienced with training, positive mood increased during exercise, whereas for UNT, it did not. This coheres with previous reports that trained individuals respond significantly more in the positive dimension during moderate- and high-intensity exercise compared to their untrained counterparts (Boutcher et al. 1997). Moreover, previous research confirms that negative affect also discriminates training status with untrained individuals demonstrating a decrease during exercise (primarily due to reductions in the ‘afraid’, ‘jittery’, ‘nervous’ and ‘scared’ categories) while trained individuals experience an increase during high-intensity exercise (Boutcher et al. 1997). A likely explanation is that unlike ATH who were familiar with intense exercise, UNT experienced a higher initial stress level that serves as a distraction which makes it difficult for them to focus on the ‘normal’ negative cognitive stimuli that are present during intense exercise (Pennebaker and Lightner 1980). The fact that PCT increased negative affect for the ATH, but not UNT, group in the present study is consistent with this possibility.

Being that the ergolytic effect of PCT has been attributed to MF (Marcora et al. 2009), it is possible that the lack of performance impairment in either group in the present study indicates that the prolonged cognitive tasks we employed did not elicit sufficient MF must be considered. We found that the reaction time and response accuracy were unaltered during the prolonged cognitive task, which involved a computer-based modified version of the incongruent Stroop colour-word task and N-back task for a continuous 30-min block. The incongruent Stroop, a cognitive task that requires inhibition of automatic responses to visual stimuli, has been used previously to induce MF prior to exercise and/or impair subsequent exercise performance. For example, despite the fact that MF was not directly measured in their study, Pageaux et al. (2014) found a reduction in self-paced 5-km TT running speed along with an increased perception of effort after 30 min of modified Stroop performance. Thirty minutes of Stroop performance also resulted in a reduction in running distance during the Yo–Yo Intermittent Recovery Test (Smith et al. 2016) along with an increased perception of effort during both Yo–Yo (Smith et al. 2016) and CWR-exercise performance (Pageaux et al. 2015). Finally, Martin et al. (2016) used the 30-min Stroop protocol when they found that athletic status determines the influence of MF on 20-min TT performance. Unlike these previous studies, however, we added the performance of the N-back task intermittently during the Stroop to reduce singular-task disengagement thereby decreasing the likelihood of a reduction in MF-inducing stimulus during the latter stages of the test (Tanaka et al. 2015). Nevertheless, it is important to recognise that the 30-min cognitive challenge that we employed might not have been long enough to achieve the psychobiologic perturbation necessary to impair subsequent exercise performance. Consequently, future research should be designed to explore the effect of longer PCTs on exercise performance in competitive athletes and untrained individuals.

In the present study, we observed a lower peak $\dot{V}{\text{O}_{2}}$ response during TT for the PCT compared to CON condition in UNT and a higher 3-min post [lactate] for the PCT compared to CON condition in ATH. However, despite these changes, which are consistent with the contention that PCT altered the metabolic challenge associated with performing the TT, exercise performance was not adversely affected in either group. Interestingly, we observed a greater HR response for untrained participants during PCT compared to the neutral-state documentary, which agrees with previous research (Pageaux et al. 2014, 2015). UNT also demonstrated a reduction in positive affect after performing the PCT. Conversely, for ATH, no difference in HR between PCT and control was observed, which likely reflects the fact that ATH show reduced autonomic and psychological stress responses compared to UNT (Rimmele et al. 2007). However, positive affect was decreased and negative affect was increased for ATH following PCT, which suggests that the intervention also alters mental state compared to the control condition in this type of individual.

During exercise, between-condition differences in ∆[HbO_2_] were consistent with the contention that UNT possess a markedly different ‘mind set’ in response to an intense exercise challenge compared to ATH. Although not without potential limitations (e.g. signal contamination due to a thermoregulatory increase in skin blood flow to the forehead; Miyazawa et al. 2013), NIRS measurements of cerebral oxygenation during exercise are highly reliable (Bhambhani et al. 2006); hence, the absence of the between-condition difference in UNT supports an interpretation that the psychological stress of unfamiliar intense exercise presents an ‘MF-like’ cognitive stimulus that PCT does not exacerbate (see above). Interestingly, the presence of these stress-related changes in cortical oxygenation in ATH might provide insight into the resistance to MF displayed by professional cyclists in the study of Martin et al. (2016). Specifically, in that study, the professional athletes improved performance throughout the 30-min Stroop and performed a 20-min TT subsequently with unchanged RPE. This implies that the athletes did not experience MF despite the demanding nature of the PCT. Conversely, in our study, ATH experienced no change in performance during the PCT. The changes in cortical activation demonstrated by ATH during exercise in our study suggest that their mental state was altered by PCT, but that this did not adversely affect exercise performance.

Unlike previous studies investigating the effect of PCT on subsequent exercise performance, we chose not to measure RPE in our study because questioning per se during intense exercise disrupts the focus of trained individuals thereby providing a stimulus that may also adversely influence cognitive state (increased negative affect due to increases in ‘hostility’ and ‘irritability’; Boutcher et al. 1997). Moreover, we used a self-regulated TT as a way to assess exercise performance with and without perturbation of the neutral mental state. TT has been employed previously in some studies investigating the effect of PCT during exercise (Pageaux et al. 2014; MacMahon et al. 2014; Martin et al. 2016) whereas others have involved CWR bouts performed until task failure (Marcora et al. 2009; Pageaux et al. 2013). We chose TTs because of their validity with respect to performance in the athletic setting and greater reliability compared to CWR tests (Currell and Jeukendrup 2008). However, we also included a 6-min severe-intensity CWR bout immediately prior to the TT so that a variety of physiological measurements could be made during an exercise task that presented the same relative challenge to all participants (i.e. sustained constant work at a work rate determined relative to each participant’s GET and peak work rates). We found that PCT did not alter the $\dot{V}{\text{O}_{2}}$, HR or blood [lactate] responses in either group during CWR, which is consistent with previous findings regarding the lack of influence of PCT on physiological factors during exercise (Marcora et al. 2009; Pageaux et al. 2015). Self-paced TT performance should be particularly sensitive to interventions that alter cognitive state. Specifically, the continuous decision-making that is present while establishing a pacing strategy likely involves significant PFC activation (Krawczyk 2002). Consistent with previous research (Pageaux et al. 2014; MacMahon et al. 2014), we found that PCT did not alter pacing strategy in either group. Furthermore, we found that both ∆[HbO_2_] and ∆[Hb_tot_] increased markedly from CWR to TT in both groups despite the fact that power output was lower during all but the final portion of the TT (i.e. a negative pacing strategy was employed). This implies that decisions regarding pacing were being established according to physiological/biomechanical factors and/or knowledge of the known endpoint of exercise rather than PFC activation per se (Billaut et al. 2010). Indeed, our NIRS data suggest that cerebral oxygenation was maintained over the course of both the severe-intensity CWR bout and TT. This coheres with the findings of Billaut et al. (2010) and is consistent with the contention that cerebral oxygenation is well preserved during severe-intensity exercise. Hence, PFC deoxygenation does not appear to hinder high-intensity exercise performance (Billaut et al. 2010; present study).

In summary, consistent with prior research on professional cyclists, we found that non-professional highly-trained individuals with a history of competition in a variety of sports do not experience an ergolytic effect of PCT at least during the performance of a 6-min cycling TT. However, a novel finding from the present study was that the TT performances of untrained participants were also not adversely impacted by a pre-exercise prolonged cognitive challenge. Although quantitatively similar responses were present in both groups, we speculate different mechanistic bases; specifically, the ability to maintain performance despite PCT in competitive athletes who are experienced with intense training and a psychobiological stress response to unfamiliar intense exercise that is similar to PCT for untrained individuals.

## Abbreviations

ACC: Anterior cingulate cortex
ATH: Competitive athletic participants
CNS: Central nervous system
CO_2_: Carbon dioxide
CON: Control condition
[cortisol]: Plasma cortisol concentration
CP: Critical power
CWR: Constant-work-rate exercise
EDTA: Ethylenediaminetetraacetic-acid
GET_met_: Metabolic rate at gas exchange threshold
[glucose]: Plasma glucose concentration
∆[HbO_2_]: Change in concentration of tissue oxyhemoglobin
∆[Hb_tot_]: Change in concentration of tissue total hemoglobin
∆[HHb]: Change in concentration of tissue deoxyhemoglobin
HR: Heart rate
INC: Incremental cycling test
[K^+^]: Plasma potassium concentration
[lactate]: Plasma lactate concentration
LH: Lithium heparin
MF: Mental fatigue
[Na^+^]: Plasma sodium concentration
NIRS: Near-infrared spectroscopy
O_2_: Oxygen
PA-R: Physical activity rating tool
PCT: Prolonged cognitive task
PFC: Prefrontal cortex
PANAS: Positive and negative affect scale
RPE: Rating of perceived exertion
TOI: Tissue oxygenation index
TT: Time trial
UNT: Untrained participants
$\dot{V}{\text{CO}}_{2}$: Rate of carbon dioxide output
$\dot{V}E$: Rate of minute ventilation
$\dot{V}{\text{O}}_{2}$: Rate of O_2_ consumption
$\dot{V}{\text{O}}_{{2{\text{peak - CWR}}}}$: Peak rate of O_2_ consumption during CWR
$\dot{V}{\text{O}}_{{2{\text{peak - INC}}}}$: Peak rate of O_2_ consumption during INC
$\dot{V}{\text{O}}_{{2{\text{peak - TT}}}}$: Peak rate of O_2_ consumption during TT
*W*_GET_: Rate of work that precipitated GET_met_
Work_TT_: Total work completed during TT
*W*_peak-INC_: Peak rate of work during INC
